# Systemic lipid dysregulation is a risk factor for macular neurodegenerative disease

**DOI:** 10.1038/s41598-020-69164-y

**Published:** 2020-07-22

**Authors:** Roberto Bonelli, Sasha M. Woods, Brendan R. E. Ansell, Tjebo F. C. Heeren, Catherine A. Egan, Kamron N. Khan, Robyn Guymer, Jennifer Trombley, Martin Friedlander, Melanie Bahlo, Marcus Fruttiger

**Affiliations:** 1 The Walter and Eliza Hall Institute of Medical Research, 1G Royal Parade, Parkville, VIC 3052 Australia; 2 Department of Medical Biology, The University of Melbourne, Melbourne, VIC 3010 Australia; 3 UCL Institute of Ophthalmology, University College London, 11-43 Bath St, London, EC1V 9EL UK; 4 Moorfields Eye Hospital NHS Foundation Trust, City Road, London, EC1 UK; 5 The Leeds Teaching Hospitals NHS Trust, St. James’s Hospital, Leeds, LS9 7TF UK; 6 Department of Surgery, Center for Eye Research Australia, Royal Victorian Eye and Ear Hospital, and Ophthalmology, 32 Gisborne St, East Melbourne, VIC 3002 Australia; 7 Lowy Medical Research Institute, La Jolla, CA USA; 8 The Scripps Research Institute, La Jolla, CA USA

**Keywords:** Data processing, Diagnostic markers, Retinal diseases, Risk factors

## Abstract

Macular Telangiectasia type 2 (MacTel) is an uncommon bilateral retinal disease, in which glial cell and photoreceptor degeneration leads to central vision loss. The causative disease mechanism is largely unknown, and no treatment is currently available. A previous study found variants in genes associated with glycine–serine metabolism (*PSPH*, *PHGDH* and *CPS1*) to be associated with MacTel, and showed low levels of glycine and serine in the serum of MacTel patients. Recently, a causative role of deoxysphingolipids in MacTel disease has been established. However, little is known about possible other metabolic dysregulation. Here we used a global metabolomics platform in a case–control study to comprehensively profile serum from 60 MacTel patients and 58 controls. Analysis of the data, using innovative computational approaches, revealed a detailed, disease-associated metabolic profile with broad changes in multiple metabolic pathways. This included alterations in the levels of several metabolites that are directly or indirectly linked to glycine–serine metabolism, further validating our previous genetic findings. We also found changes unrelated to PSPH, PHGDH and CPS1 activity. Most pronounced, levels of several lipid groups were altered, with increased phosphatidylethanolamines being the most affected lipid group. Assessing correlations between different metabolites across our samples revealed putative functional connections. Correlations between phosphatidylethanolamines and sphingomyelin, and glycine–serine and sphingomyelin, observed in controls, were reduced in MacTel patients, suggesting metabolic re-wiring of sphingomyelin metabolism in MacTel patients. Our findings provide novel insights into metabolic changes associated with MacTel and implicate altered lipid metabolism as a contributor to this retinal neurodegenerative disease.

## Introduction

Macular telangiectasia type 2 (MacTel) is an uncommon, bilateral neurodegenerative retinal disease affecting between 0.004 and 0.1% of the population^1,2^. It is characterized by alterations of the macular capillary network and neurosensory atrophy beginning temporal to the fovea, eventually affecting the so-called “MacTel area”; an oval area approximately 3 mm across the temporal-nasal axis and 2 mm across the superior-inferior axis centred on the fovea and of similar size in all patients^3^. Symptoms typically start in the 5th and 6th decade of life, most commonly with reading difficulties and distortions^3–6^. The pathogenic mechanism of this disease is still not fully understood, but post-mortem histopathological studies show abnormalities in the retinal pigment epithelium (RPE) throughout the retina^7^ and a complete loss of Müller cells specifically in the MacTel area; which also contains some regions of photoreceptor loss^8^. A recent phase 2 clinical trial with an ocular implant secreting ciliary neurotrophic factor showed promising results in delaying disease progression, but no evidence of recovery^9^. No other therapeutic treatments are currently available.

Several factors suggest that MacTel has a substantial genetic component. The disease occurs bilaterally and is heritable based on studies of monozygotic twins, siblings and families^3,10–13^. In a Genome-Wide Association Analysis (GWAS) for MacTel we previously identified five genetic loci associated with the disease^14^; of which four are associated with glycine and serine abundance in serum^15–17^. We also observed lower glycine and serine levels in the serum of MacTel patients relative to controls^14^. One of the several metabolic pathways in which serine is need, is the biosynthesis of sphinganine, which forms the backbone of all sphingolipids^18^. Low serine can lead to the formation of atypical sphingolipids (deoxysphingolipids). We recently found that low serine in the serum of MacTel patients correlates with increased levels of deoxysphingolipids^19^, which are known to have cytotoxic properties^18^. However, how deoxysphingolipids (and low glycine/serine) contribute to the MacTel disease mechanism remains unclear; in particular, which factors contribute to the local retinal specificity of the disease in otherwise healthy individuals. The degree to which other metabolites may contribute to MacTel requires further investigation.

To comprehensively characterise the metabolomic profile of MacTel patients, we analysed hundreds of different serum metabolites using a global metabolomics platform. The vast data captured by this technology requires advanced data processing and statistical analysis^20,21^. Here we employed advanced statistical methodologies^20,22,23^, to characterise the impact of reduced glycine and serine in a broad metabolic context, and identify novel metabolic pathways associated with MacTel.

## Materials and methods

### Study participants and serum collection

From our MacTel Natural History and Observation Study (NHOS) blood serum was available from 60 random patients. Control samples were collected from 58 unrelated individuals without MacTel (confirmed by ophthalmic examination by an ophthalmologist using fundoscopy and optical coherence tomography). Control patients were selectively included to achieve similar demographics as in the MacTel patient cohort regarding age, gender, diabetic status and ethnicity. However, we did not achieve perfect symmetry between the two cohorts within the timeframe of this study (Table S1), and therefore used multiple regression modelling in our downstream statistical analysis to correct for potential confounders effects (see “Statistical analysis” section).

The number of samples in our study was based on previous experience by Metabolon (Durham, USA) regarding effect size of serum metabolomic analysis on the analytical platform used. Samples (patients and controls) were collected at different clinical sites (Moorfields Eye Hospital London, UK; Royal Victorian Eye and Ear Hospital and Ophthalmology, Melbourne, Australia; Scripps Health Facility, Scripps Clinic Torrey Pines, La Jolla, USA, and St. James’ Hospital, Leeds, UK) according to a standardised protocol. All individuals fasted overnight, and blood was taken before noon. Around 5 ml of blood was collected in a clot activating vacutainer tube (Vacutainer Plastic SST II Advance Tube with Gold Hemogard Closure, Becton Dickinson), left at room temperature for 30 min and then centrifuged for 5 min at 1,200*g*. The supernatant was collected, frozen and stored at − 80 °C.

### Metabolite measurements

Serum metabolites were measured by Metabolon (Durham, USA). Briefly, this involved initial protein precipitation with methanol under vigorous shaking for 2 min (Glen Mills GenoGrinder 2000) followed by centrifugation. The resulting extract was divided into five fractions: two for analysis by two separate reverse phase (RP)/UPLC–MS/MS methods with positive ion mode electrospray ionization (ESI); one for analysis by RP/UPLC-MS/MS with negative ion mode ESI; and one for analysis by HILIC/UPLC-MS/MS with negative ion mode ESI. Raw data was extracted, peak-identified and QC processed using Metabolon’s hardware and software. Compounds were identified by comparison to library entries of purified standards and peaks were quantified using the area-under-the-curve technique, providing relative abundances of 946 individual metabolites.

### Metabolomics data processing

Missing values for some metabolites indicated levels below the detection limit of the Metabolon platform. For this reason, missing abundances were imputed with the minimum value for each metabolite following Metabolon’s standard imputation protocol. In subsequent analyses, we discovered how imputed minimum values for each metabolite were driving a substantial amount of variance captured by principal components (PCs). Because of this, we decided to discard 194 metabolites which had more than 20% of their total values missing from the analysis; using the previously proposed “80% rule”^24,25^. Metabolomics missingness in controls correlated with missingness in patients, which ensured compatibility between patients and control (Fig. S2) and ensured that missingness would not confound the disease signal. The average missingness per subject was 13.3% with SD 1.5%. Boxplot outlier detection was performed on the distribution of missing values among subjects: this analysis did not detect any outlier subjects for missingness, and no particular subject was excluded for excessive amount of missingness.

A total of 738 metabolites passed quality control steps and were further analysed. We used the R software package limma^26^ to further quantile normalise the data between samples and ensure correction for any batch effects not captured by the initial area-under-the-curve normalization. This software package was initially developed for gene expression analysis but has been cross-purposed for metabolite analyses^27–29^. To interrogate whether the heterogeneity in the remaining metabolites was driven by the diversity between subject demographics we visually inspected the first two principal components against all available covariates. Visual exploration of principal components plots ensured that no unwanted variation was observable after the scaling and normalisation step (Fig. S3). Lastly, we scaled each metabolite to have zero mean and unit standard deviation to ensure comparability for each specific metabolomic result.

### Statistical analysis

Differences in demographics between patients and controls were tested using Chi-squared tests (for dichotomous demographics) and *t* tests with Welch correction for unequal variances (for continuous variables). We tested each metabolite against disease status using the limma package for the statistical software, R^26^. This package has been widely used in gene expression study and is based on an empirical Bayes approach; whose properties for the analysis of both microarray data and RNAseq data have been described elsewhere^26,30^. For this study we used a false discovery rate cut-off of 5%. To this end, we corrected each *p* value for false discovery rate by applying the Benjamini–Hochberg *p* value correction procedure on the nominal uncorrected *p* values using the R function p.adjust^31^. All metabolites with a corrected *p* value less than the false discovery rate cut off of 0.05 were considered as significant. Each model corrected for all available covariates which included sex at birth, age, diabetes status, ethnicity and BMI. A clustered heatmap of significant metabolites is presented in (Fig. S4). To test for any residual bias due to sample ethnic imbalances we have compared the results using all samples with the results using Caucasian samples only (Fig. S5) and found virtually equivalent results.

After defining the groups, we tested for enrichment by using a popular tool for gene enrichment pathway analysis—ROAST—available in the limma package. The properties and details of this tool have been discussed elsewhere^32^. We corrected for false discovery rate by correcting each *p* value using the Benjamini–Hochberg procedure. All groups with a corrected *p* value of less than 0.05 were considered as significant. To account for groups in which metabolites were changed in opposite directions, we represented the abundance of grouped metabolites as a single value (the first principal component) and performed differential expression analysis using the same method applied to individual metabolites.

To test for differential co-abundance, we firstly calculated the correlation between pairs of metabolites within patients and within controls. However, metabolomics correlation can be confounded by a set of external factors. To account for this, we firstly residualised all metabolomics profiles for each covariate jointly. This was achieved by regressing each metabolite on the set of covariates and MacTel status using a linear regression model and sequentially extracting the model residuals. This approach ensured that no correlation between metabolite was either created or masked by the covariates. After residualisation, we excluded from the analysis all those metabolites pairs for which the absolute value of their correlation was less than 0.5. We then tested for differential co-abundance between pairs of metabolites similarly to previous work^33^ by using the Fisher R to Z transformation as defined in the Psych package^34^. We corrected for false discovery rate by correcting each retained *p* value using Benjamini–Hochberg procedure. Given the very high number of correlation pairs tested and the reduced sample size, all pairs with a corrected *p* value less than 0.1 were considered as significantly differentially co-abundant. Following the definition of previous work on gene expression by Jiang et al.^33^ we explored the hypothesis that specific groups might present an abnormal amount of differential co-abundance. Accordingly, we discarded all metabolites belonging to the xenobiotic super classification from this analysis. To test the hypothesis of groups enriched with differential co-abundance, we used a binomial test. The number of successes was the number of significant differential co-abundant pairs which contained a metabolite in that group. The number of tests was the total number of co-abundant pairs tested which contained a metabolite belonging to that group. Lastly, the probability parameter was defined as the ratio between all significantly differentially co-abundant pairs in the dataset—161—and all tested pairs 19.127 (p_parameter = 0.008). We corrected for false discovery rate by correcting each *p* value using Benjamini–Hochberg procedure. All metabolites with a corrected *p* value less than 0.05 were considered as enriched.

### Ethics approval and consent to participate

All experiments were conducted according to the principles expressed in the Declaration of Helsinki. All participants gave informed consent and the study was approved by the Research Ethics Office Bromley, UK (study number 05/Q0504/101).

## Results

### Metabolite levels

**Figure 1:**
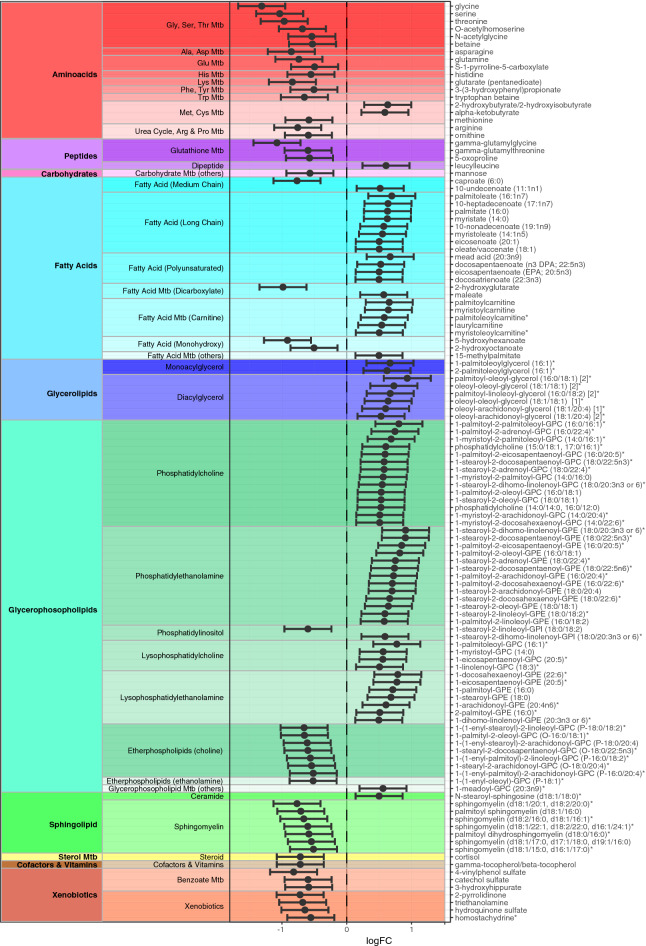
Visual representation of all 121 significantly differentially abundant metabolites, comparing MacTel patients against controls. Each row represents a metabolite. The x-axis represents the LogFC. Negative LogFC values indicate reduced metabolite levels in MacTel patients compared to controls, and positive LogFC values indicate increased levels in MacTel patients. The model results are presented as dots indicating the estimated logFC with 95% confidence interval bars. Metabolites are divided into coloured blocks by their metabolic group. *Mtb* metabolism.

We conducted an untargeted global metabolomic analysis (Metabolon, Inc.) of serum from 60 extensively phenotyped MacTel patients and 58 healthy controls, measuring the relative levels of a total of 946 known metabolites, of which 738 survived quality control. The control cohort had similar demographics as the patient cohort (Table S1), and statistical tests (see “Materials and methods” section) revealed no significant differences between them, regarding average age (*p* = 0.06), gender (*p* = 0.14), body mass index (BMI, *p* = 0.34) and—since diabetes is a comorbidity of MacTel^35,36^—diabetic status (*p* = 0.22). However, there was a difference regarding ethnicity status between cases and controls (*p* = 0.009). Nevertheless, we used normalisation and multivariate regression strategies to correct for all available covariates (age, gender, diabetes status, ethnicity, BMI and collection site, as described in “Materials and methods” section). The fully normalised dataset (Table S2) was then analysed for differential abundance of individual metabolites in patients versus controls. This revealed 49 metabolites with significantly lower serum concentrations in MacTel patients compared to controls, and 72 with elevated serum concentrations (*p* < 0.05, corrected for false discovery rate, FDR) (Fig. 1).

**Figure 2:**
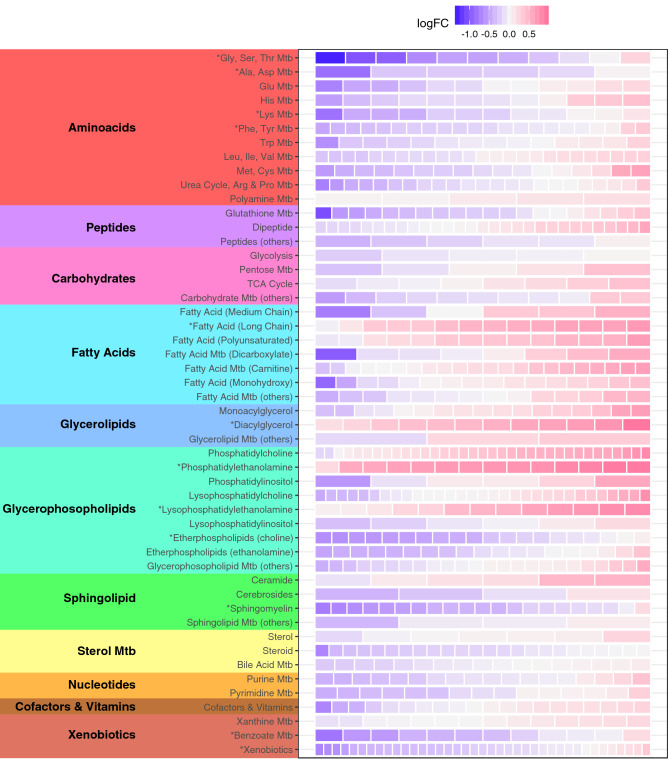
Changes in abundance of 738 metabolites across the 50 metabolic groups. Each row represents a metabolic group. Significantly enriched metabolic groups are labelled with * for *p* < 0.05 (corrected for FDR). Each group row is composed of blocks representing metabolites contained in the group. The colour of each block represents the Log_2_ Fold-Change (logFC) of that metabolite comparing patients against controls. The colour blue represents depletion and magenta represents increased abundance. *Mtb* metabolism.

Glycine and serine were the first and third most depleted metabolites in MacTel patients (log(2) fold changes (logFC) of − 1.31 and − 1.03, respectively). This agrees with our recent GWAS^14^, which also showed reduced glycine/serine concentrations in MacTel patients, using a subset of the samples presented here. The second and fourth most changed metabolites were gamma-glutamylglycine and alpha-ketoglutarate (logFC of − 1.08 and − 0.98, respectively), both of which are linked to glycine–serine metabolism (see “Discussion” section). However, we also found many changed metabolites belonging to other metabolism groups (Figs. 1, 2). For instance, we found reduced levels of arginine (logFC = − 0.76), ornithine (logFC = − 0.59) and guanidinoacetate (logFC = − 0.47), which—together with glycine—are needed for creatine biosynthesis (see “Discussion” section). Similarly, methionine (logFC = − 0.58) and betaine (logFC = − 0.53), which are linked to cysteine–methionine metabolism (see “Discussion” section), were reduced in MacTel patients. In contrast, the majority of measured lipids were increased. Furthermore, of the 121 significantly changed metabolites, 88 were lipids (73%), whilst the total dataset of 738 metabolites contained 45% lipids, suggesting a disproportional impact on lipid metabolism in MacTel.

**Figure 3:**
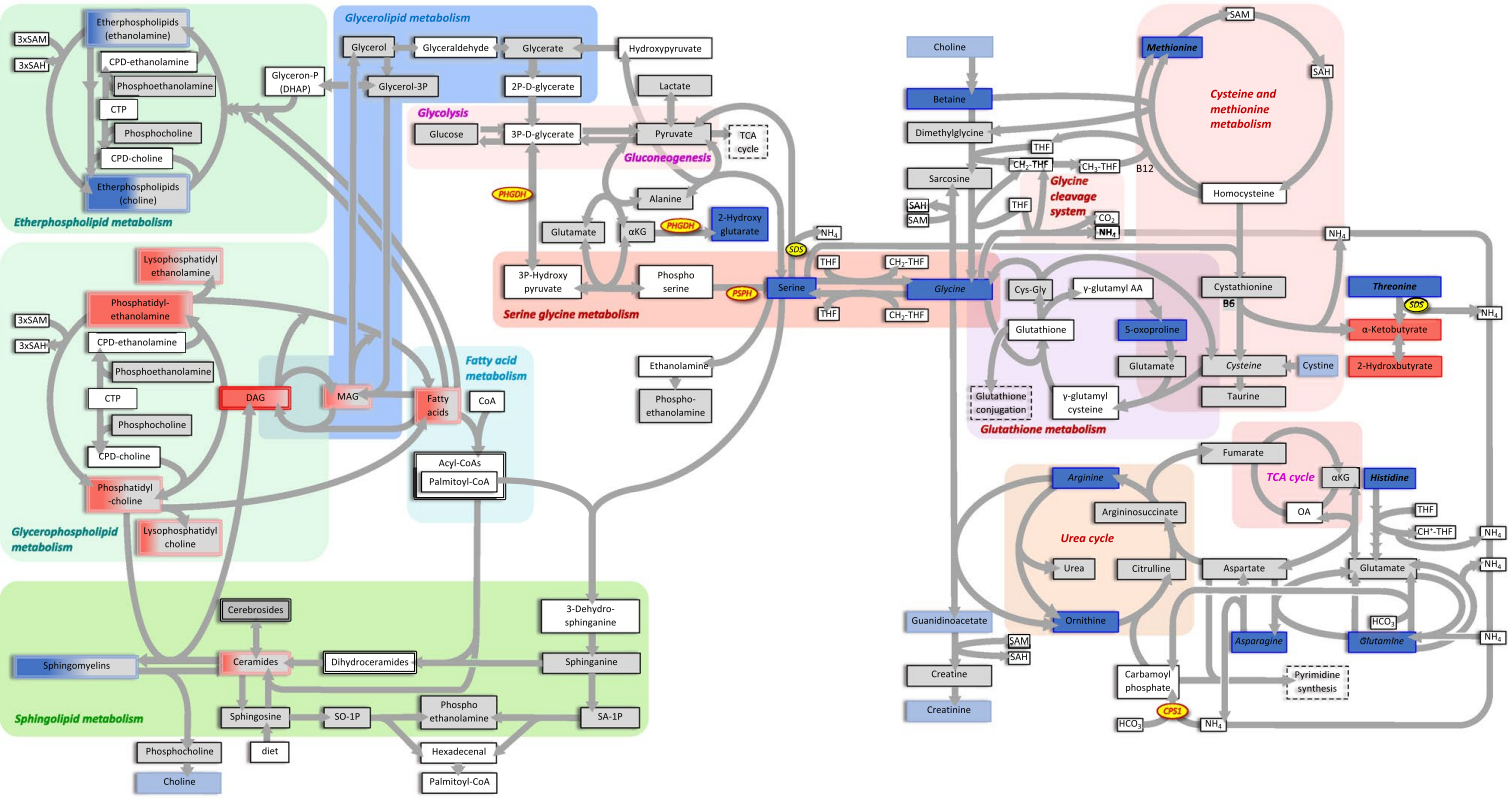
Graphical overview of the key metabolic pathways that were affected in MacTel patients. Metabolites in blue were reduced in patients (*p* < 0.05 in dark blue, 0.05 < *p* < 0.1 in light blue). Metabolites in red were increased in MacTel patients (*p* < 0.05 in dark red, 0.05 < *p* < 0.1 in light red). Grey indicates no change between patients and controls, and metabolites on a white background were not measured. Double borders around metabolites indicates multiple metabolites within a group. The gene names of enzymes mentioned in the text are in yellow ovals. Note the generally reduced metabolite levels in glycine–serine and adjacent metabolic pathways, and generally increased levels in glycerophospholipid metabolism.

To fully assess whether the observed changes affected specific metabolic pathways, we divided all metabolites into 50 functional groups—largely reflecting pathways assigned by the Kyoto Encyclopaedia of Genes and Genomes (KEGG) database—and tested (described in “Materials and methods” section) whether any groups presented enrichment of differential abundance (Fig. 3, Table S3).

The most differentially abundant metabolite group in MacTel patients was glycine–serine–threonine metabolism (*p* = 2.5E−5), with 7 of 11 measured metabolites depleted. The second most significantly different group was the phosphatidylethanolamines (*p* = 8.8E−5), with 13 out of 14 metabolites upregulated in MacTel patients. Other differentially abundant groups included long chain fatty acids (increased, *p* = 0.02) and diacylglycerols (increased, *p* = 0.012), as well as changes in alanine-asparagine (reduced, *p* = 0.012), methionine–cysteine (mixed, PC *p* = 0.018) and benzoate metabolism (reduced *p* = 0.002). We further detected several pronounced lipid group differences including increases in lysophosphatidylethanolamines (*p* = 0.0054), diacylglycerols (*p* = 0.012), monoacylglycerols (*p* = 0.463) and long chain fatty acids (*p* = 0.020) (Table S3). Although the majority of lipids were increased in MacTel patients, etherlipids with a choline headgroup were markedly reduced (*p* = 0.020). A second group of lipids showing a reverse trend of general lipid increases in MacTel patients were the sphingomyelins (*p* = 0.012), where 20 of the 21 measured species were reduced (7 of which were significant; Fig. 1).

### Correlations between metabolites

Having established that the abundance of several metabolites was altered in MacTel patients we investigated how metabolites correlated with each other (co-abundance) across patients and controls. This technique can reveal molecular interactions that change in the context of disease^37^ and can inform potentially dysregulated biochemical mechanisms^33,38,39^. Differential correlation testing was limited to those metabolite pairs which were significantly correlated after correction for multiple comparisons in either patients or controls. Whereas most correlations between metabolites were similar across both groups (Fig. S1), three were significantly different in MacTel patients compared to the controls (Table S4). Orotate and orotidine were correlated in patients (r = 0.73) but not controls (r = 0.02; *p* = 0.001); and the correlation between xanthine and orotidine was negative in controls (r = − 0.43) but positive in patients (r = 0.43; *p* = 0.001). These metabolites all function within pyrimidine metabolism. However, closer inspection of these results revealed a potential xenobiotic confound, driven by four patients with elevated oxypurinol levels (not shown), likely due to Allopurinol drug exposure, which is known to disturb pyrimidine metabolism.

**Figure 4:**
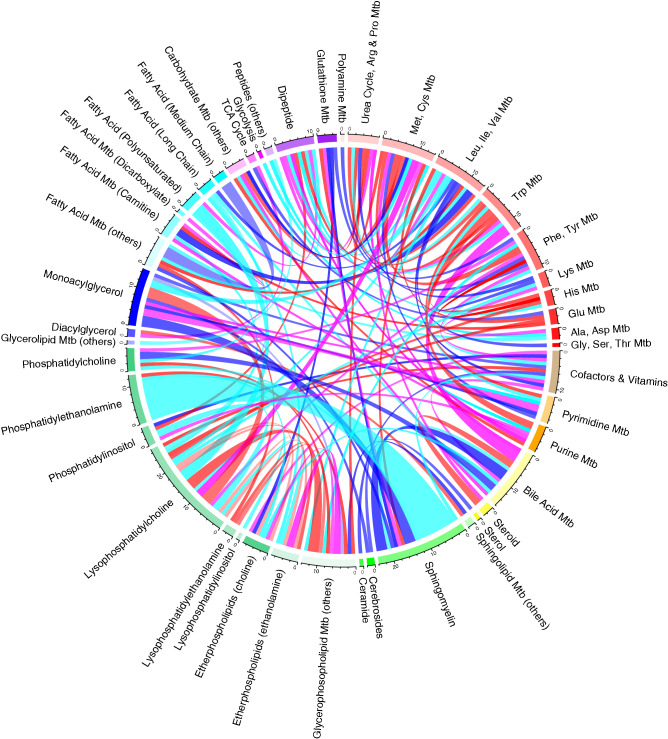
Circos plot displaying 152 significantly differentially co-abundant metabolite pairs across 46 metabolic groups. Differential co-abundance between metabolic groups is represented by a line connecting the relevant groups. Thickness indicates the number of significant differential co-abundances. Correlations that were lost in patients are displayed in blue (positive correlation in controls and correlation lost in patients) and cyan (negative correlation in controls and correlation lost in patients). Newly formed connections in patients are presented in red (positive correlation in patients not observed in controls) and magenta (negative correlation in patients not observed in controls). The transparency of the lines represents correlation magnitude (more transparency = lower magnitude). Note that connections involving the sphingomyelin group are most strongly suppressed in patients. *Mtb* metabolism.

**Figure 5:**
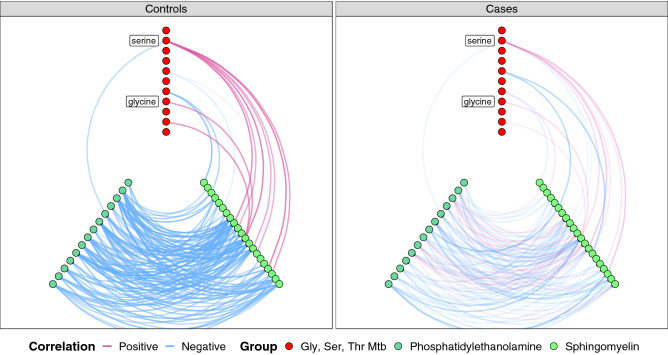
Hive plots comparing co-abundance between metabolites in the sphingomyelin (13), phosphatidylethanolamine (14) and glycine–serine metabolism (11) groups in controls and MacTel patients. Specific metabolites are represented by circles. Co-abundance correlation between metabolites is represented by a line. Line transparency represents the correlation magnitude. Red lines represent positive correlations while blue lines represent negative correlations. Note that the majority of connections evident in controls are lost or markedly reduced in MacTel patients. *Mtb *metabolism.

We additionally tested for enrichment in differential co-abundance at the metabolite group (pathway) level. Tests of enrichment for differential co-expression were performed using a binomial framework^33^. Of 45 metabolite groups, seven were enriched in differentially correlated metabolite groups in patients compared to controls (*p* < 0.05; Fig. 4, Table S5). Among these, methionine–cysteine metabolism and the sphingomyelin group overlapped with our metabolite abundance results. The sphingomyelin group exhibited the most homogeneous pattern of differential co-abundance, characterised by a unique enrichment in disrupted connections. Specifically, differential correlation between sphingomyelins and the most differentially abundant groups—phosphatidylethanolamines and glycine–serine–threonine pathway metabolites—were driving this result. Positive correlations between the sphingomyelins and serine–glycine pathway metabolites, observed in controls, were lost in MacTel patients; while negative correlations between sphingomyelins and the phosphatidylethanolamines, were reduced or lost in patients (Fig. 5). This striking result represents disruption of the normal metabolic links between serine, phosphatidylethanolamines and sphingomyelins in sphingolipid metabolism of MacTel patients.

## Discussion

### Data analysis tools

Careful data collection and demographic balance between cases and control by demographic information and batch control is an important part of any metabolomics study. As a perfect study design is rarely achieved for rare diseases such MacTel, statistical approaches must be used to address any potential confounding arising from imperfections. As such, this study uses a wide variety of statistical tools and techniques to reach maximal discovery power while ensuring reproducibility as well as additional sensitivity analyses to assess the key results presented in the paper.

Isolating relevant signals in metabolomics data requires advanced statistical methods. For this study we used limma software^26^, widely used in gene expression analysis, to normalize for confounding factors, construct sample quality weights and perform multivariate modelling. Additionally, by using an empirical Bayesian framework that “borrows” information between metabolites, the software ensures maximal discovery power even for extremely variable metabolites^26,30^. To extend our finding beyond simple biomarkers, we performed enrichment analysis using ROAST^32^ which takes into account metabolite abundances rather than summary statistics^30,32^. In addition, we conducted semi-supervised principal components analysis to assess significance in groups containing metabolites with mixed correlations—which might have been missed by ROAST—as well as exploring metabolic network changes through differential co-abundance analysis. Although the computational tools we employed here have so far not been commonly used for metabolomics data analysis, in this study we demonstrate that their deployment in the field of metabolomics can be extremely useful and powerful.

### Genetic variants in PSPH, PHGDH and CPS1 deeply impact metabolic profiles in MacTel patients

Our previous GWAS^14^ identified MacTel disease risk-associated single nucleotide polymorphisms (SNPs) within the three genes, *PSPH* (encoding phosphoserine phosphatase), *PHGDH* (encoding phosphoglycerate dehydrogenase) and *CPS1* (encoding carbamoyl-phosphate synthase), which are all known to contribute to glycine–serine metabolism. PSPH and PHGDH catalyse consecutive steps in the serine biosynthesis pathway (Fig. 2), and mutations in *PSPH* are associated with reduced plasma serine levels^40^. Similarly, SNP rs477992 reduces *PHGDH* transcription^41^ and serine plasma levels^16,17^. This matches well with the clear reductions in glycine and serine in MacTel patient serum we are presenting here. Furthermore, PHGDH is also known to convert alpha-ketoglutarate to 2-hydroxyglutarate^42,43^. While the former was only marginally increased in patient serum (logFC = 0.40), the latter was strongly depleted (logFC = − 0.98), further supporting a likely PHGDH defect in MacTel patients. Serine depletion, caused by dysfunctional PSPH and PHGDH, increases conversion of glycine to serine (Fig. 2)^44^, explaining the reduced glycine levels, at least in part.

Another likely mechanism contributing to low glycine in MacTel patients is based on the activity of CPS1, which is connected to the urea cycle and creatine biosynthesis (Fig. 2). CPS1 converts ammonia and bicarbonate into carbamoyl phosphate, which then feeds the urea cycle. The C allele of SNP rs715 (located in the 3′ UTR of *CPS1*; estimated MAF = 0.24) has been found in a previous independent study to be strongly associated with increased glycine serum levels^16^. *CPS1* is also implicated, via GWAS studies, in modulating creatine^16^, arginine and ornithine levels^45^. These effects are based on the role of CPS1 in creatine biosynthesis, which requires urea metabolites. In a key intermediary step, glycine and arginine are converted to guanidinoacetate and ornithine (Fig. 2), all four of which we found to be reduced in MacTel serum (logFC = − 1.31, logFC = − 0.76, logFC = − 0.46 and logFC = − 0.59, respectively). Reduced CPS1 activity slows the urea cycle and the linked guanidino-acetate production. This likely results in reduced glycine consumption and may explain the protective role of rs715(C) in MacTel^14^.

Mutations in *CPS1* might also explain the depleted threonine levels (logFC = − 0.96) in our MacTel patients (Figs. 1, 2) since a previous GWAS has linked *CPS1* with threonine plasma levels^45^. However, the biochemical connections between CPS1 and threonine are not clear. Whilst most mammals can directly convert glycine to threonine, the enzyme responsible for this reaction (threonine aldolase) has lost function in humans^46^, making threonine an essential amino acid. Alternatively, it is conceivable that microbiome influences may contribute to glycine–threonine conversion^47^.

In addition, several further metabolites linked to *CPS1* via GWAS^45,48^ were reduced in our MacTel samples (asparagine, logFC = − 0.85; glutamine, logFC = − 0.74 and betaine, logFC = − 0.53). Asparagine and glutamine have been linked to CPS1 by a GWAS^45^, matching the correlations found in our study (glycine–asparagine, r = 0.65 and glycine–glutamine, r = 0.51, in controls). As glutamine is needed to create intracellular asparagine, which in turn is needed for serine uptake^49^, depletion of these metabolites—as observed in our study—is likely to additionally contribute to the low serine availability observed in MacTel. However, the correlation between these metabolites was not changed in MacTel patients compared to controls (glycine–asparagine, r = 0.63 and glycine–glutamine, r = 0.56). Of interest, there was a trend towards reduced correlation between glycine and betaine (r = 0.56 in controls, r = 0.16 in MacTel patients), but this change did not attain statistical significance (*p* = 0.22).

In addition, we observed a trend towards changed correlations between serine and pyruvate, which rose from r = − 0.16 in controls to r = 0.39 in patients. Although the change did not reach statistical significance (*p* = 0.13), it is interesting that these two metabolites are linked via serine dehydratase (SDS), which converts serine to pyruvate (Fig. 2). An increased correlation between serine and pyruvate possibly indicates a more pronounced usage of this pathway in MacTel patients, which would reduce serine levels. Of note, SDS can also degrade threonine to α-ketobutyrate and ammonia (Fig. 2), which might be reflected by the increased α-ketobutyrate levels in MacTel serum due to a potential increase of SDS activity in our patients.

### Changes in metabolites related to cysteine/methionine metabolism imply oxidative stress and phospholipid species bias in MacTel

GWAS has also linked *CPS1* to the metabolites betaine, choline and homocysteine^48,50^, which are all relevant for cysteine/methionine metabolism (Fig. 2). In MacTel patients, betaine and choline were both reduced (logFC = − 0.53, logFC = − 0.46, respectively), which might relate to the fact that methionine was also lower in MacTel patients (logFC = − 0.58). The strong correlations we observed between methionine and asparagine (r = 0.73 in controls, r = 0.69 in MacTel patients) and between methionine and threonine (r = 0.61 in controls, r = 0.74 in MacTel patients) support the notion of a potential involvement of CPS1. However, the mechanism by which CPS1 could influence methionine levels is not known.

Methionine is an essential amino acid and can be recycled from homocysteine via two different pathways. One depends on betaine (as mentioned above, low in MacTel patients), while the other requires 5,10-methylenetetrahydrofolate (CH2-THF in Fig. 2), which was not directly measured in our study, but is likely to be reduced given its close metabolic links to serine and glycine^51^. Additionally, low choline and betaine observed in MacTel patients increases the reliance of the methionine cycle on one-carbon metabolism, adding a further demand on glycine. Furthermore, histidine—also used to add one carbon to tetrahydrofolate—was also reduced in MacTel patients (logFC = − 0.55). Together, these findings suggest lower methionine cycle capacity in MacTel patients compared to controls.

In this context it is interesting that MacTel patients exhibited increased concentrations of alpha-ketobutyrate (logFC = 0.59) and 2-hydroxybutyrate (logFC = 0.63), which may relate either to increased threonine degradation or to increased glutathione production (Fig. 2). Possible reasons for the latter are increased oxidative stress or detoxification in the liver^52^. Increased glutathione synthesis consumes serine and glycine. Furthermore, it also limits the supply of cysteine, diverting homocysteine away from the transmethylation pathway towards glutathione synthesis, leading to a stressed transmethylation pathway.

A key product of the methionine cycle is S-adenosylmethionine (SAM), required for transmethylation reactions. The conversion of phosphatidylethanolamine to phosphatidylcholine requires three SAM molecules and is, therefore, particularly affected by methionine cycle limitations. This strongly agrees with the aforementioned increase of phosphatidylethanolamines found in MacTel patients. Although phosphatidylcholine levels were nominally elevated (logFC = 0.38, p = 0.076), the phosphatidylethanolamine increase was much more pronounced (logFC = 0.81, p = 8.8e−05), apparent in nearly all measured phosphatidylethanolamine species (Fig. 2, Table S3). Interestingly, ether lipids with an ethanolamine head group were less severely reduced, mirroring the shift in the ethanolamine/choline ratio mentioned here.

The lower abundance of methionine observed in MacTel patients may be connected to changes in both glutathione and phosphatidylcholine synthesis. As a relative lack of methionine may impair glutathione synthesis, this might expose MacTel patients to a higher oxidative stress load. Additionally, the methionine derivative SAM provides the methyl group substrates required to produce choline from ethanolamine. In fact, we found increased phosphatidylethanolamine in MacTel patients, suggesting an imbalance in the phosphatidylethanolamine/phosphatidylcholine ratio. Further, substantial reduction in choline relative to ethanolamine etherphosphoilipids in patients is also consistent with methionine depletion.

### Lipid dysregulation is a novel disease risk factor for MacTel

Several lipid groups were significantly changed in MacTel patients; with increased phosphatidylethanolamines, lysophosphatidylethanolamines and diacylglycerols, and decreased sphingomyelins (Table S3). These changes cannot be linked (directly or indirectly) to the activity of PSPH, PHGDH and CPS1, based on current understanding of human metabolic pathways. It is, therefore, plausible that the observed lipid dysregulation in MacTel patients represents a novel MacTel risk factor, in addition to the previously identified risk factors related to glycine–serine metabolism. The abnormal lipid levels in MacTel patients may be caused by as yet unidentified genetic risks, as well as by environmental or dietary influences.

### Sphingomyelins link dysregulated lipids and serine metabolism

Differential co-abundance analysis identified sphingomyelins as an important metabolic group for MacTel. Not only were sphingomyelins depleted in MacTel, but metabolic connections—indicated by correlations of metabolites—between this group with the glycine–serine metabolism group, and the phosphatidylethanolamine group, were lost (Fig. 5). Although serine was depleted in MacTel patients, phosphatidylethanolamines and their fatty acid constituents were enriched. This striking change in correlation is particularly interesting in context of our recent finding that deoxysphingolipids are an important component in MacTel^19^. How exactly deoxysphingolipids contribute to retinal pathology in MacTel is not fully understood yet, but an intriguing possibility is that part of their toxic properties might be mediated by dysregulation of sphingomyelins, which are well known to differentially regulate apoptosis and autophagy^53^. In this context it is also interesting that ceramides were partially enriched in MacTel patients (2 out of 6 measured). Ceramides can act as pro-apoptotic signalling molecules, and it has been shown that blocking ceramide biosynthesis prevents photoreceptor cell death in a mouse model of retinitis pigmentosa^54^.

### MacTel metabolic dysregulation might affect type 2 diabetes risk

Previous studies have observed a high prevalence of type 2 diabetes among MacTel patients^35,36^. However, the mechanism explaining this association is not understood. We therefore roughly matched for diabetic status in our MacTel and control cohorts (Table S1), and used a computational approach (described in “Materials and methods” section) to correct for any remaining imbalances. Despite this, our study identified an intriguing overlap between MacTel metabolites and previously described metabolic phenotypes associated with type 2 diabetes^55–57^. For instance, MacTel patients displayed increased fatty acids, diacylglycerols and phosphatidylethanolamines, as well as reduced etherlipids, glycine and glutamine, a similar pattern to that observed in type 2 diabetes metabolomics studies, hinting at potentially shared mechanistic links between MacTel and diabetes. Additionally, the recent finding of increased deoxy-sphingolipids in MacTel patients also aligns with previous findings of deoxy-sphingolipids abundance among Type 2 Diabetes patients^58–60^, further strengthening our observation of possible metabolic overlap between the two diseases.

### Using serum metabolomics to study a complex eye disease

MacTel is a genetically complex and moderately clinically heterogeneous disease involving focal degeneration of retinal glia and photoreceptors, and vascular damage of the central retina (macula). MacTel heterogeneity may arise from a complex mechanism impacting multiple biological pathways. Our previous study^14^ provided initial evidence that the disease was associated with global alterations of metabolism, determined in part by complex genetic contributions. To explore this hypothesis, we comprehensively compared the metabolome of MacTel patients to that of healthy controls. Metabolomics is an emerging field and has been recognised as a powerful tool in ophthalmological research^58,59^. In this study, we used serum samples, although MacTel is a retinal disease not characterised by any known systemic pathology. This may appear counterintuitive, as retinal metabolism is considered to be isolated from the peripheral blood circulation due to the blood-retina barrier. However, MacTel is associated with systemic hyperglycaemia^35,36^, and metabolomics profiles of blood samples have been used to study other retinal diseases, identifying changes as potential metabolic biomarkers or interrogating broad dysregulations^60–63^. Furthermore, serum samples are more readily available than retina samples in people with the disease, and metabolomics platforms for analysis are readily and commercially available. We recently also showed in mice that systemic serine and glycine changes (induced by removing serine and glycine from the diet) were clearly reflected in the retina^19^. Finally, we note that although our study could not profit from a validation set, our findings are strengthened by the clear alignment with our previous publications on MacTel genetics^14^ and some metabolic features, such as reduced glycine and serine^19^.

It is important to keep in mind that, although our study has identified systemic risk factors for the disease, the metabolic changes in MacTel retina are so far unknown. It is also unknown by what route metabolic changes might affect the retina. It is possible that the underlying genotypes that cause the systemic changes—for example due to altered liver metabolism – affect the retina indirectly via the circulation. On the other hand, it is also possible that the genes that contribute to the systemic changes may have independent functions in the retina where they directly contribute to the disease.

## Conclusion

In summary, in this study we have revealed putative functional relationships between multiple metabolite groups and MacTel disease using global metabolomic analyses. Several of the changes we observed in this study confirm our previously identified genetic MacTel findings where we implicated *PSPH*, *PHGDH* and *CPS1*; however, many other changes are novel and represent novel risk factors for this disease. Our study provides not only a foundation for future genetic and experimental analyses of MacTel pathobiology, but also serves as a template for the use of computational approaches to global metabolomics data to investigate diseases with complex aetiologies.

## Supplementary information

Supplementary Information 1.

Supplementary Information 2.

## Data Availability

Raw data not included in this published article is included as supplementary information.
