# Supplementary material for: Systemic lipid dysregulation is a risk factor for macular neurodegenerative disease
Source: Sci Rep. 2020 Jul 22;10:12165. doi: 10.1038/s41598-020-69164-y (PMC7376024; doi:10.1038/s41598-020-69164-y)
Supplement: Supplementary file 1 — Supplementary Information 1. [file 41598_2020_69164_MOESM1_ESM.docx]

Systemic lipid dysregulation is a risk factor for macular neurodegenerative disease.

Roberto Bonelli, Sasha M Woods, Brendan R. E. Ansell, Tjebo FC Heeren, Catherine A. Egan, Kamron N. Khan, Robyn Guymer, Jennifer Trombley, Martin Friedlander, Melanie Bahlo, Marcus Fruttiger.

**Supplementary information**

# Supplementary Figures

**Figure S1**

**
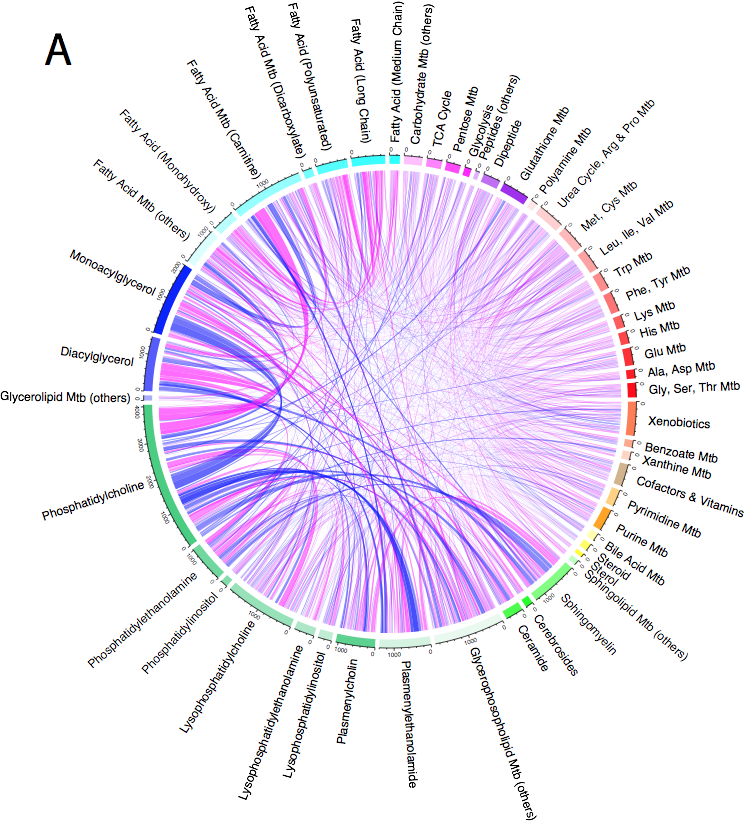
**

**Fig. S1A:** Circos plot visualising all co-abundance between metabolites in controls in different metabolic groups. Each connection between metabolic groups is represented by a line. Thickness indicates the number of co-abundances greater in absolute value to 0.5. Magenta lines represent positive correlation while blue represent negatives. The transparency of the lines indicates a correlation. Both circos include correlations that were greater in absolute value to 0.5 in either cases or controls. Missing connections indicates controls-specific or case-specific correlations. Note how in cases circos most of the correlations branching out from the sphingomyelins are missing.


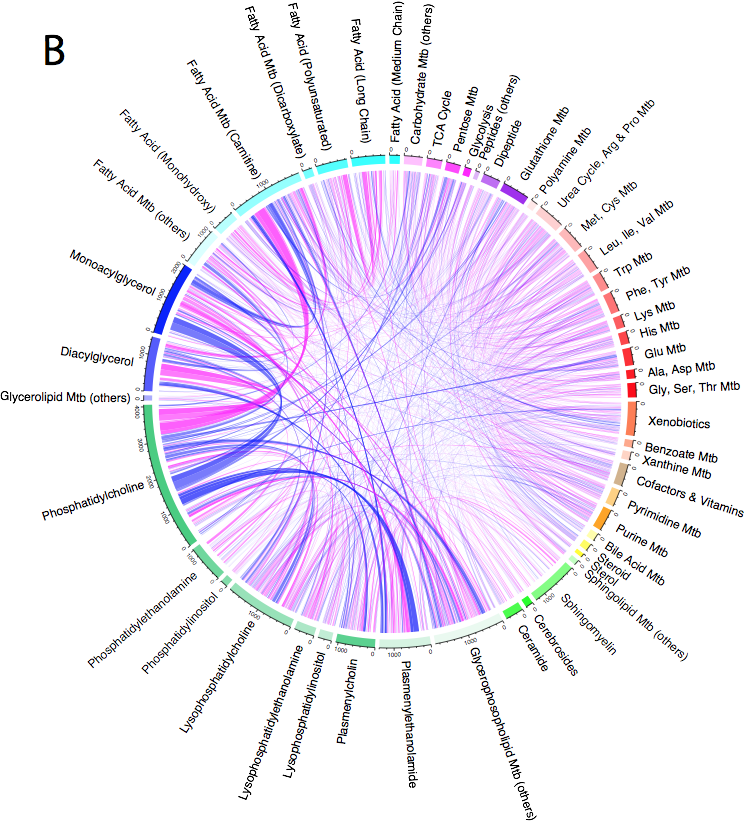


**Fig. S1B:** Circos plot visualising all co-abundance between metabolites in cases in different metabolic groups. Each connection between metabolic groups is represented by a line. Thickness indicates the number of co-abundances greater in absolute value to 0.5. Magenta lines represent positive correlation while blue represent negatives. The transparency of the lines indicates a correlation. Both circos include correlations that were greater in absolute value to 0.5 in either cases or controls. Missing connections indicates controls-specific or case-specific correlations. Note how in cases circos most of the correlations branching out from the sphingomyelins are missing.

**Figure S2**


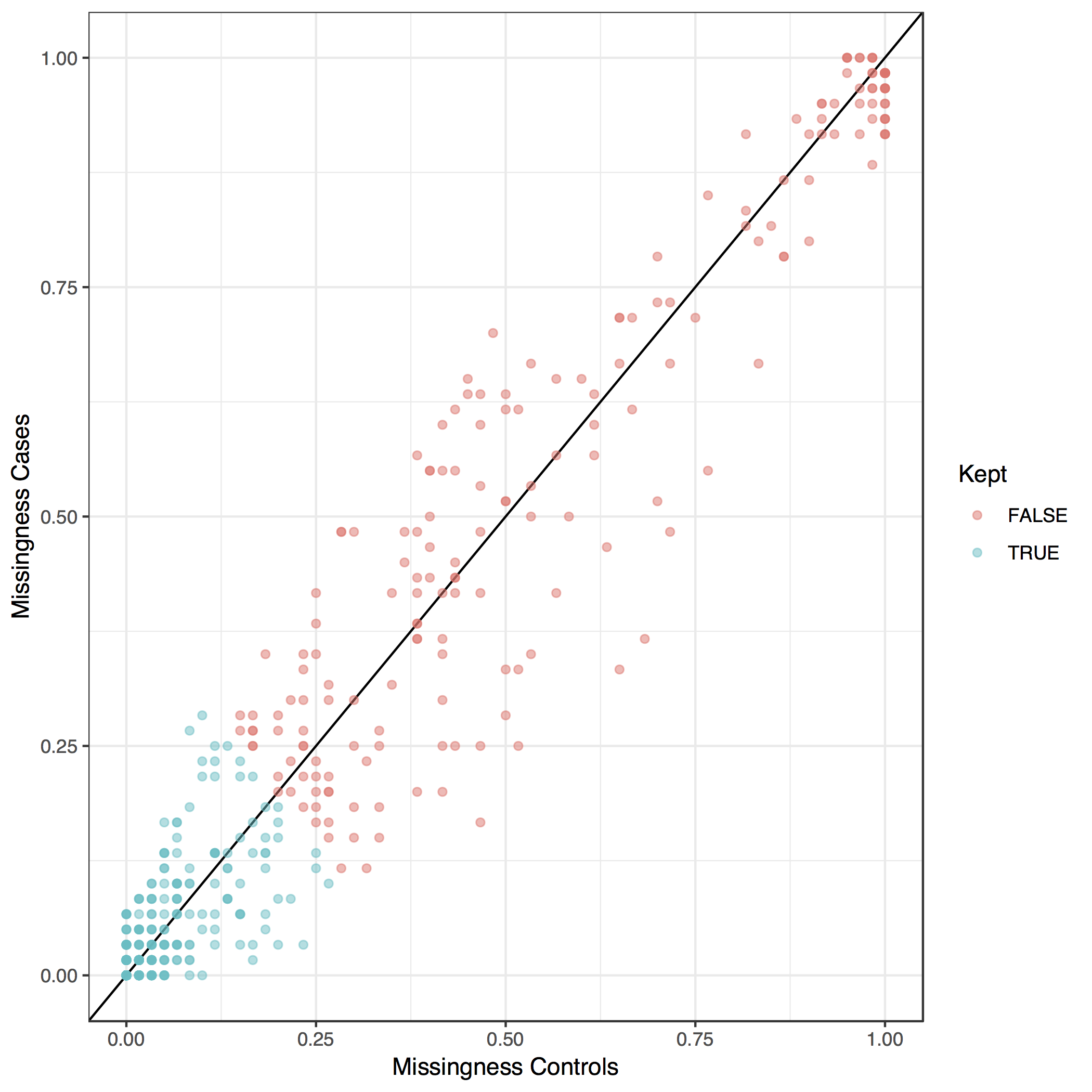


**Fig. S2:** Metabolomics missingness rate comparison. In this figure, each metabolite is a point. Missingness of each metabolite is indicated by the x-axis in controls and by y-axis for cases. Metabolites with average missingness less than of 0.2 were kept in the study. Darker points indicated the presence of multiple metabolites overlapping on each other. The black line represents the identity line. Note how missingness rate between cases and controls heavily correlates.

**Figure S3**

**A**


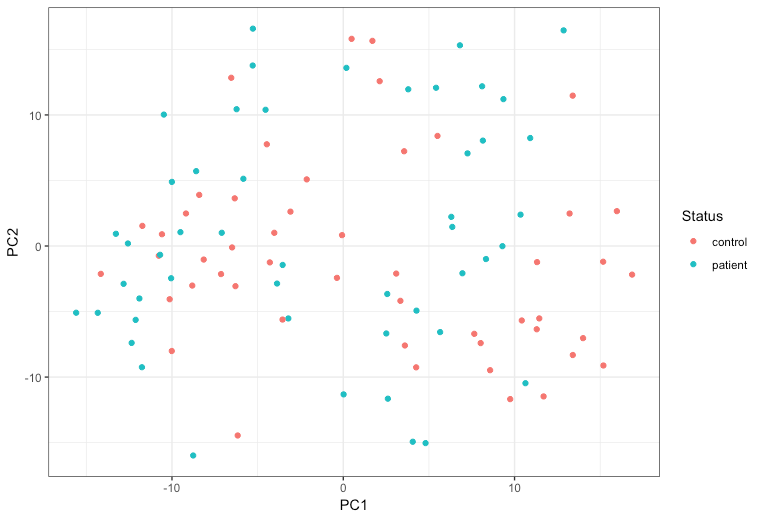


**B**


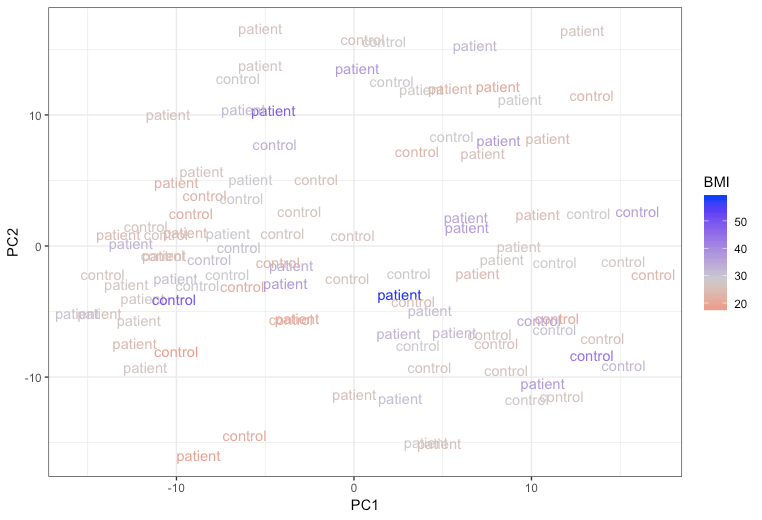


**C**


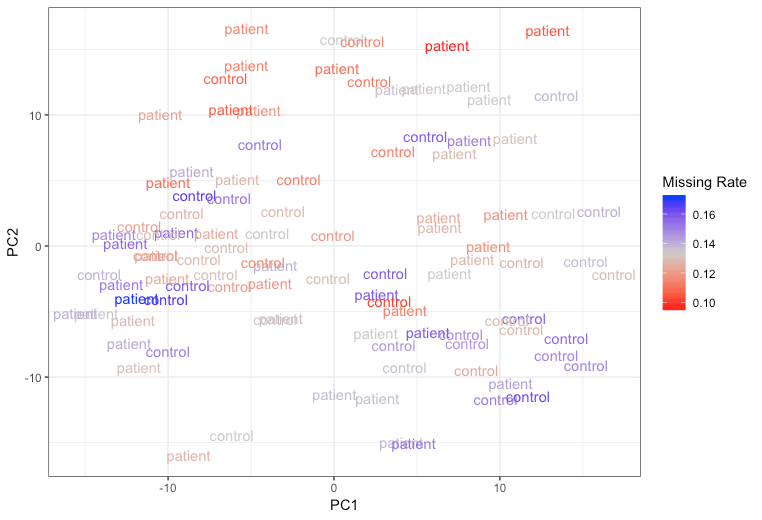


**D**


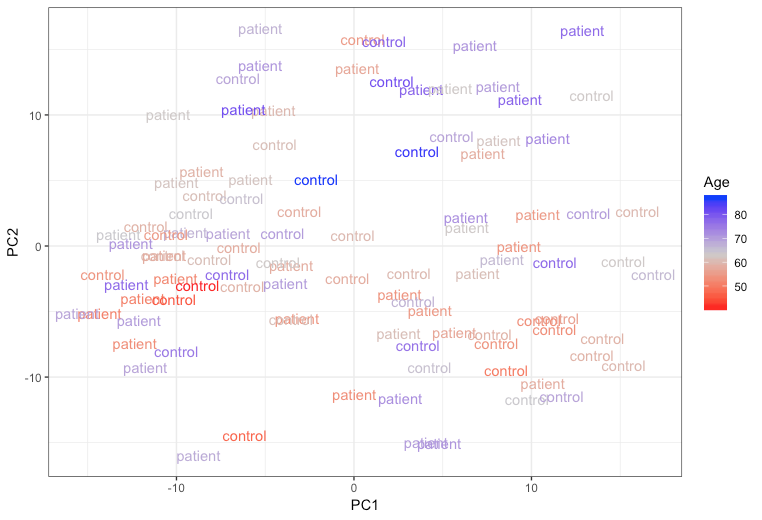


**E**


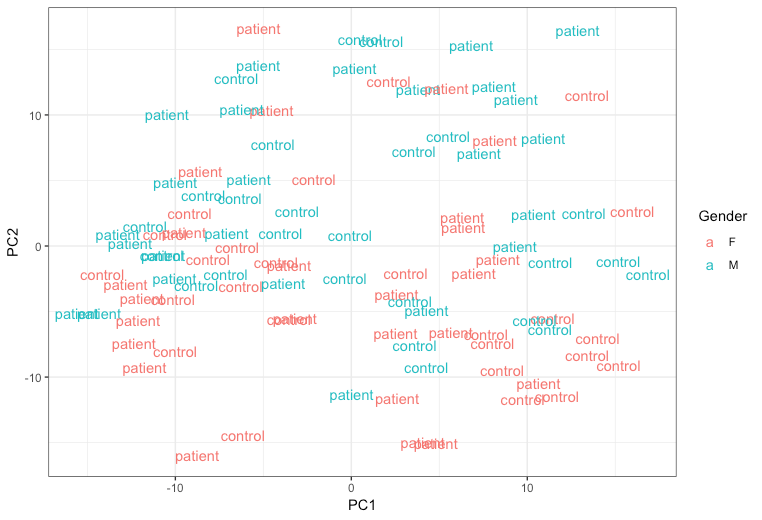


**F**


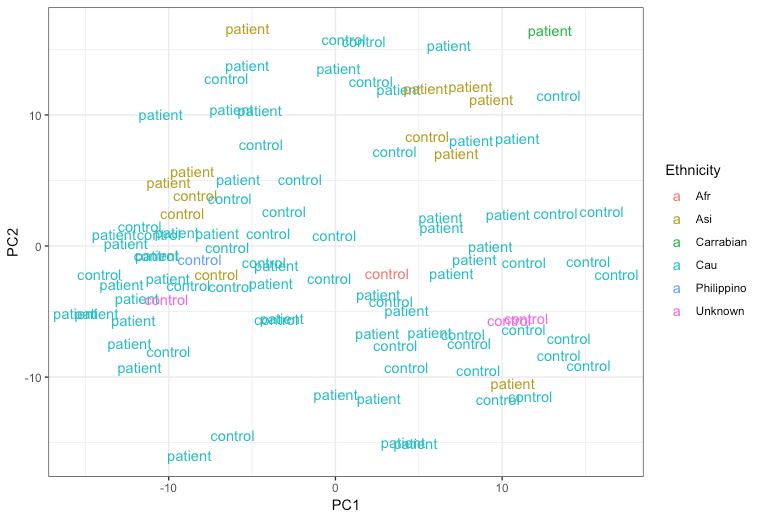


**G**


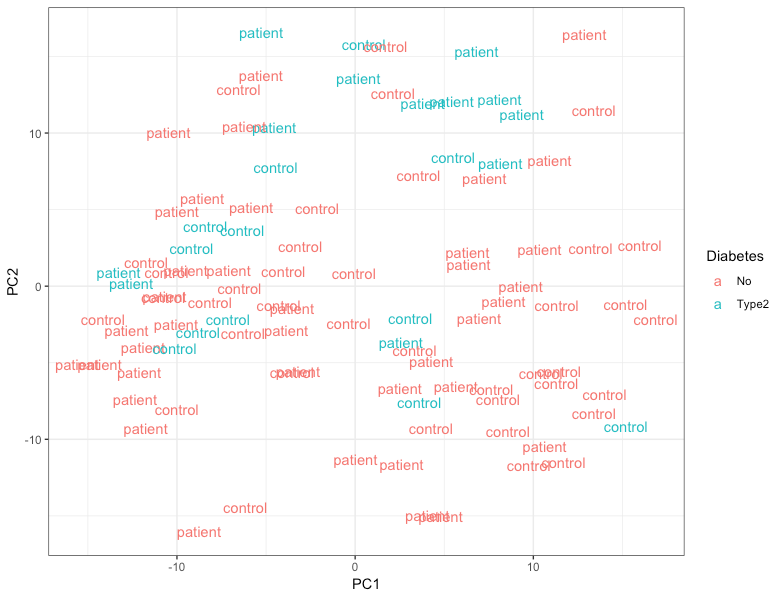


**Fig. S3:** Principal components plot displaying the 100 subjects heterogeneity. Each dot or string represents a particular subject in the study. Dots or strings are coloured accordingly to a specific covariate recorded in the study: (A) Case-control status, (B) BMI, (C) metabolomic missing rate, (D) Age, (E) Gender, (F) Ethnicity, and (G) Diabetes status. Note how there is no strong clustering in the first two principal components for each covariate indicating no strong confounding effect.

**Figure S4**


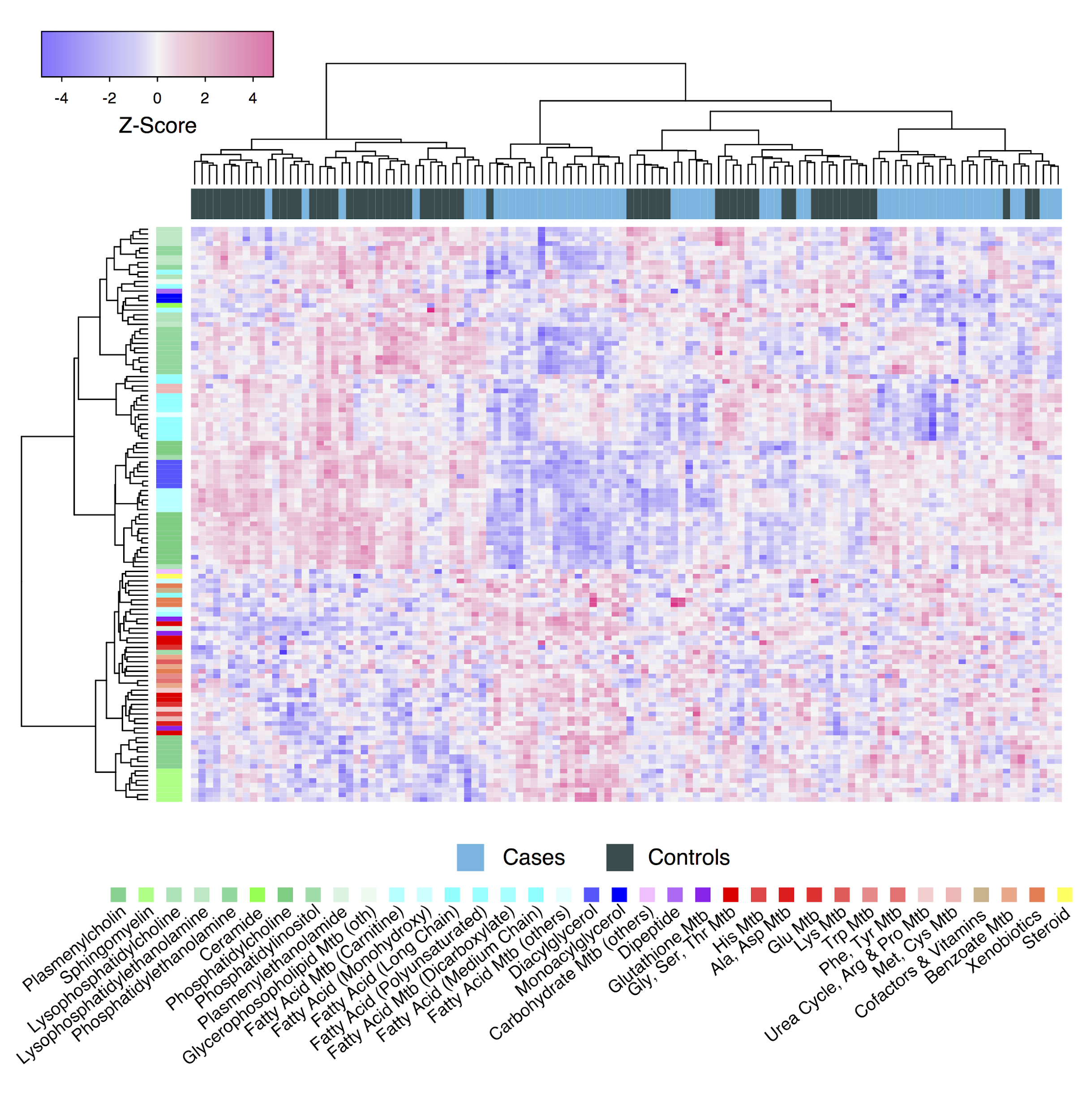


**Fig. S4:** Heatmap of displaying normalised metabolomics abundances for all significant metabolites. Each row is a metabolite and each column is a subject. Rows are named according to the metabolic group defined in the study. Columns are coloured according to disease status.

**Figure S5**

**A**

**
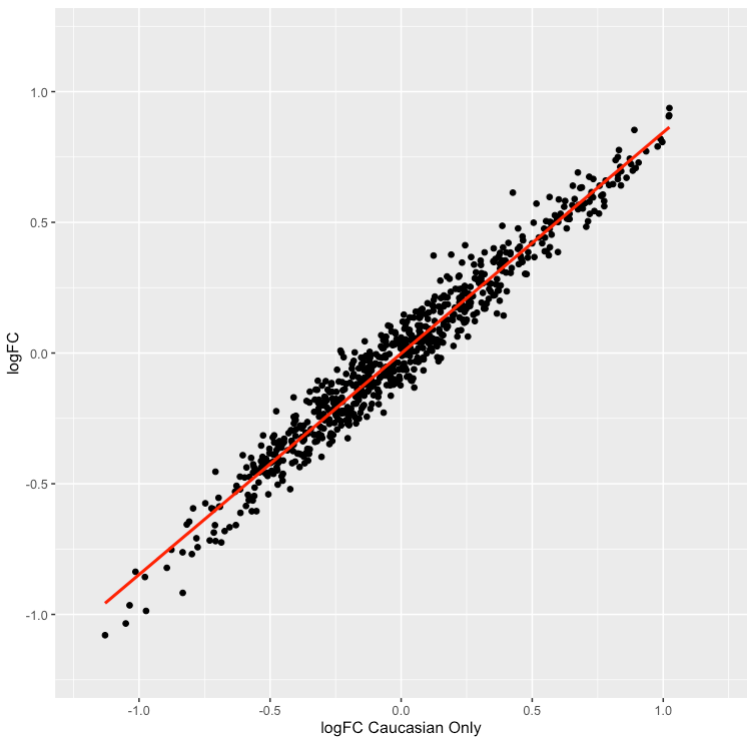
**

**B**

**
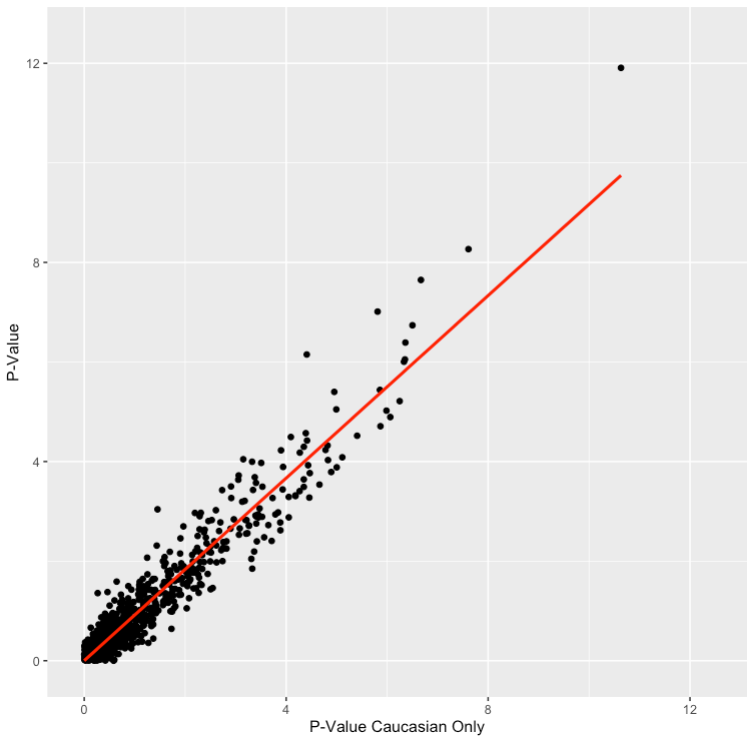
**

**Fig. S5:** Quality control analysis comparing metabolites logFC (A) and P-Value (B) in all samples and in Caucasian only to assess potential residual effect of ethnic background differences. (A) Metabolites log-fold changes correlations coefficient r=0.98. (B) Metabolites -log10 P-values correlations coefficient r=0.98.

# Supplementary Tables

Tables S1, S3, S3, S4, and S5 are provided in the suppplementary_tables.xlsx file. A description of all tables is provided below.

**Table S1:** Sample demographics description.

**Table S2:** Metabolomics differentially abundance results. Metabolites names are available in the column “BIOCHEMICAL”. Nominal p-values are indicated in column p-value while FDR corrected p-value can be found in the column “FDR”. Metabolites are sorted by significance level.

**Table S3:** Enrichment analysis results for all groups and PC analysis results. Enrichment analysis directionality of the group is indicated by the column “Direction”. Nominal p-values are indicated in column Enrichment p-value while Enrichment FDR corrected p-value can be found in the column “FDR”. Groups are sorted by significance level. Group principal component analysis results are presented as PC logFC indicating the magnitude of the effect of disease status on group PC abundance. Nominal p-values are indicated in column PC p-value. Multiple testing corrected p-values are presented as PC FDR.

**Table S4:** List of significant differentially co-abundant metabolite pairs. This list contains all metabolite pairs that were considered differentially co-abundant. Correlation in controls is displayed in column “Cor Controls” while correlation in cases is displayed in column “Cor Cases”. Nominal p-values are indicated in column p-value while FDR corrected p-value can be found in the column “FDR”. Each pairs is available in columns “BIOCHEMICAL1” and “BIOCHEMICAL2”. To provide an easier exploration of the results, each pair is presented twice with the metabolite in each pair being displayed one time in both columns. The results the table are sorted by metabolites names in “BIOCHEMICAL1”. For example, to explore all the metabolites changing their correlation with serine in cases compared to controls, simply scroll the table until serine is in BIOCHEMICAL1”, then look at the metabolites names in the column “BIOCHEMICAL2”.

**Table S5**: List of all groups significantly enriched with differential co-abundance. This list contains all those groups that were considered significantly enriched with differential co-abundance. The column “Significant pairs” is the number of significant differentially co-abundant pairs containing at least one metabolite belonging to that group. The column “Tested pairs” is the number of all considered metabolites pairs (a correlation in either cases or controls greater than 0.5 in absolute value) containing at least one metabolite belonging to that group. Nominal p-values are indicated in column P-value while FDR corrected p-value can be found in the column “FDR”
